# Ebola Virus: The need to take some preventive measures

**DOI:** 10.12669/pjms.312.7154

**Published:** 2015

**Authors:** Mohammad Dilawar Jan, Rafay Ahmed, Urooj Zafar

**Affiliations:** 1 Mohammad Dilawar Jan, Student, Final Year MBBS, Dow University of Health Sciences, Karachi, Pakistan; 2 Rafay Ahmed, Student, Final Year MBBS, Dow University of Health Sciences, Karachi, Pakistan; 3 Urooj Zafar, Student, Final Year MBBS, Dow University of Health Sciences, Karachi, Pakistan

Ebola Virus Disease (EVD) is a viral infectious disease. This virus has caused a number of outbreaks since its discovery in 1976 but the current West African outbreak is the biggest of all.^1^,^2^ Currently five countries in the West Africa are affected by this deadly disease viz, Liberia, Sierra Leone, Guinea, Nigeria and Mali. Mali is the newest to join the list of the affected African nations. Meanwhile the virus has also travelled to other nations in the world by the effected travelers. This spread of the virus by unchecked travels across the globe is currently one of the emerging threats. In the past, outbreaks of the Ebola virus were limited to the isolated villages in Africa. But this current outbreak is out of its limits mainly because it is spreading to the major cities of the affected countries from where it is finding its way out through air and sea travels. Some international doctors and nurses mainly American and Spanish, who flew to West Africa to help patients recover, were also infected by this virus. They were flown back to their countries and they recovered from the disease.

It should be kept in mind that there is no cure for this deadly disease until now and recovery is only based on symptomatic treatment with best medical facilities. Three experimental drugs “ZMapp” (an experimental biopharmaceutical drug comprising three chimeric monoclonal antibodies)^3^, “TKM-Ebola” (an RNA interference drug)^4^ and Brincidofovir (an antiviral drug)^5^ have been approved by the FDA to be used for American people who are infected but these drugs are not subjected to human trials. Two vaccines have been chosen as candidates for Phase 1 Clinical Trials, “cAd3-ZEBOV” (developed by GlaxoSmithKline in collaboration with the US National Institute of Allergy and Infectious Diseases) and “rVSV-ZEBOV” (developed by the Public Health Agency of Canada).^6^ The American vaccine “cAd3-ZEBOV” has cleared safety tests and it does produce strong immune reaction against the virus in healthy subjects. These results have been published in New England Journal of Medicine.^7^

Michael T. Osterholm wrote an article “What We’re Afraid to Say About Ebola” in The New York Times (Dated: Sept 11, 2014) where he quotes “What happens when an infected person yet to become ill travels by plane to Lagos, Nairobi, Kinshasa or Mogadishu — or even Karachi, Jakarta, Mexico City or Dhaka?”.^8^ His fear was not wrong at that time because those of us who have some idea about the principles of public health in terms of infectious diseases epidemics and outbreaks, such large metropolitan cities like mentioned above are a fruitful ground for the virus to reside itself and ultimately spread itself in the community based on the its characteristics of transmissibility like reproductive number, generation time, serial time etc.

This fear of ours, unfortunately, recently got true when a middle aged Pakistani national resident in Liberia flew out of its capital to Karachi.^9^ Officials say that he was not febrile when he was in Liberia nor when he got aboard the flight but he was found to have a fever of 103 when he landed in Karachi. This patient was been moved to the isolation unit in a public sector tertiary care hospital (JPMC).

Upon further probing it was found that another suspected case of Ebola, died in a local hospital in Faisalabad.^10^ Patient’s blood sample reports showed that he died of Dengue fever and Hepatitis C. This man flew out of Togolese Republic, a West African country but not affected by Ebola currently. This incident clearly shows the amount of panic this disease arrival has caused. Recently another suspected case was admitted in PIMS Hospital, Islamabad.^11^ Fearfully this number would rise with time.

In any case of such an outbreak, the biggest problems are the lack of communication and the difference of risk perception between the scientific community, especially medical professionals, and the public. To deal with this, WHO outbreak communication guidelines should be followed.^12^ These guidelines help in communication with public during an outbreak. The 5 point guidelines include Trust, Announcing Early, Transparency, The Public and Planning. The officials must try to create a sense of trust so that what they tell the people about the outbreak they understand it and have trust in them. The concerned authorities should announce early every new case or any new development regarding the outbreak. This information should be highly transparent without any disguise or lie. The beliefs of the public and further planning should be done accordingly and at a fast pace.

When we look at the problems in dealing with new outbreaks, we clearly feel that the medical personnel in our country lack the expertise to handle such infected patients. This is a common problem being faced by almost all developing nations, one hurdle also being that different pathogens require different levels of biosafety. For Ebola BSL (Bio-safety Level) 4 protection is required.^13^ The medical personnel in the affected West African countries are being trained and helped by the volunteers of international organizations like Medecins San Frontieres (Doctors Without Borders), Partners in Health, The Centers for Disease Control (CDC) and USAID.^14^ Such training is lacking in our country and our government should focus on the training of medical personnel.

To curb the spread of outbreak itself, the government should act upon a number of guidelines such as Guiding Principles for International Outbreak Alert and Response presented by the Global Outbreak Alert and Response Network (GOARN) of WHO^15^ and the Crisis & Emergency Risk Communication (CERC) by the CDC.^16^ These guidelines also put forward how the government should inform about the infected cases to international organizations such as WHO or CDC. Such measures help in the sharing of information among international institutions and laboratories working towards the common goal of curtailing this outbreak.

The entry points to our country whether these are the airports or the seaports, are lacking in the technical equipment that are needed to screen the suspected cases. Recently Karachi seaport authority (KPT) has implemented checking measures which includes the ship’s captain to provide a list of all those city harbors which he has docked but no screening for the ships crew.^17^ At Karachi international airport, the passengers in bound are not being checked for their body temperatures. A forehead strip was used to check the temperature of the suspected case and this was only done after the officials noticed his Health Card.^9^ If we ponder on this, temperature of all passengers should be recorded by infrared thermometers. This type of instrument allows temperature recording without touching the person. This non-contact technique is of particular importance because Ebola is transmitted by bodily secretions like blood, sweat, saliva etc.^18^ Another concern is to gather information about suspected case’s contacts and their surveillance.

It is also important that we understand the difference between isolation and quarantine. The isolation applies to persons who are known to be ill with a contagious disease and quarantine applies to those who have been exposed to a contagious disease but who may or may not become ill.^19^ Quarantine is also based on the incubation period of the disease. The incubation period is the time interval from infection with the virus to onset of symptoms. For Ebola it is two to 21 days. So the suggested quarantine time for Ebola is 21 days.^20^ The quarantined persons should then have symptomatic and serologic surveillance.

Ebola virus has four strains: Bundibugyo, Sudan, Taï Forest, Zaire and Reston. Zaire strain is responsible for the current West African outbreak while Reston strain is not thought to cause disease in humans.^21^ Ebola is a single stranded RNA virus. RNA viruses have a high mutation rate because viral RNA polymerases lack the proof reading ability that DNA polymerases have. This is the reason why it has always been difficult to create vaccines against RNA viruses.^22^ Many of the recent emerging and re-emerging infectious diseases outbreaks are caused by RNA viruses such as Corona viruses (MERS and SARS), Poliovirus, Norwalk virus, Yellow fever virus, West Nile virus, Dengue fever virus, Chikungunya virus, Marburg virus, Nipah virus, Hendra virus, Lassa virus, Hantavirus, Crimean-Congo hemorrhagic fever virus and Influenza viruses (Avian/Bird flu, Swine flu). So another concern is that this virus could get mutated and become transmissible through the air. This concern is over the fact that a Canadian researcher’s team in 2012 found out that one of the strains of Ebola virus (Zaire strain) can be transmitted by respiratory route from pigs to monkeys. This route of transmission could also happen in humans if the virus mutates itself to do so.^23^ We should not forget how SARS in 2002, Swine Flu (H1N1) in 2009 and Highly Pathogenic Avian Influenza “HPAI” (H5N1) spread rapidly through air.^24^,^25^ Most of these viruses including Ebola cause zoonoses i.e. disease that transmits from one animal species to other, particularly to humans. Bats, pigs and birds act as major reservoirs for these viruses.^26^ The Pakistan government has made a four member Rapid Response Team for Ebola patients which includes doctors from different institutions of the country but this step is not enough. It is suggested that government should make a National Ebola Steering Committee headed by the Prime Minister just as one for the Polio eradication is made. Such a step is the utmost need of time because this is a new threat for which no vaccine or drug is available neither the country has expertise in this regard. The Sindh government has issued a notification for making 5 isolation wards for Ebola patients in a small teaching hospital (which was once a TB sanatorium) of one of the public sector medical university. It is suggested that such isolation wards be made outside the city as early as possible.

In the end we would like to highlight again the fact that our medical personnel lack the facilities and expertise to handle such a deadly pathogen. It is therefore necessary that government take steps for their training, purchase of Personal Protective Equipment (PPE), purchase of infrared thermometers, proper transport of suspected cases and building of new isolation wards outside the city.
